# Supplementary material for: Effect of the Proximity to the Quintero-Puchuncaví Industrial Zone on Compounds Isolated from Baccharis macraei Hook. & Arn: Their Antioxidant and Cytotoxic Activity
Source: Int J Mol Sci. 2024 May 30;25(11):5993. doi: 10.3390/ijms25115993 (PMC11172710; doi:10.3390/ijms25115993)
Supplement: Supplementary file 1 [file ijms-25-05993-s001.zip › ijms-3006900-supplementary.pdf]

Supplementary Materials

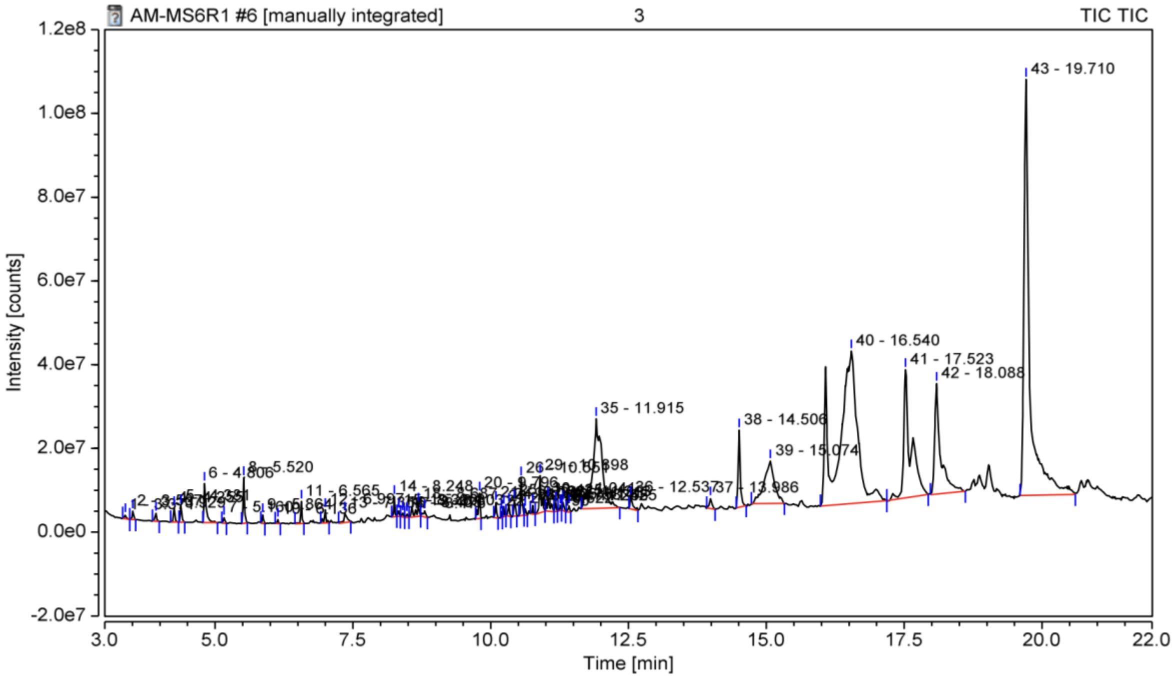

Figure S1: GC-MS chromatograms of ethyl acetate extract of *B. macraei* leaf from the far zone.

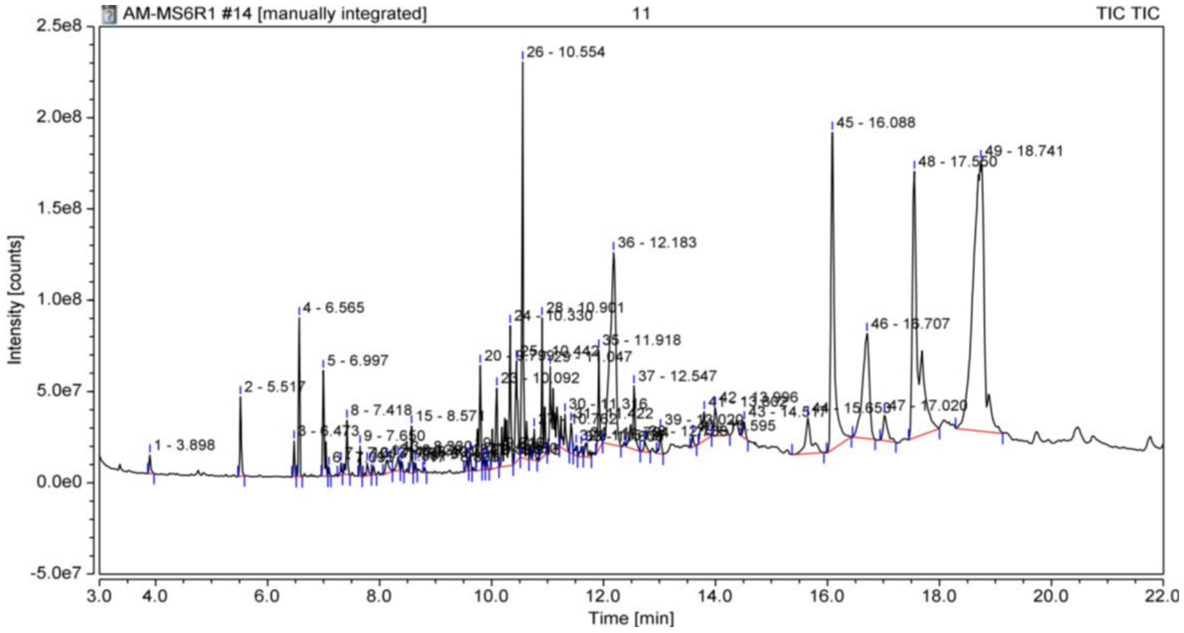

Figure S2: GC-MS chromatograms of ethyl acetate extract of *B. macraei* leaf from the Near zone.

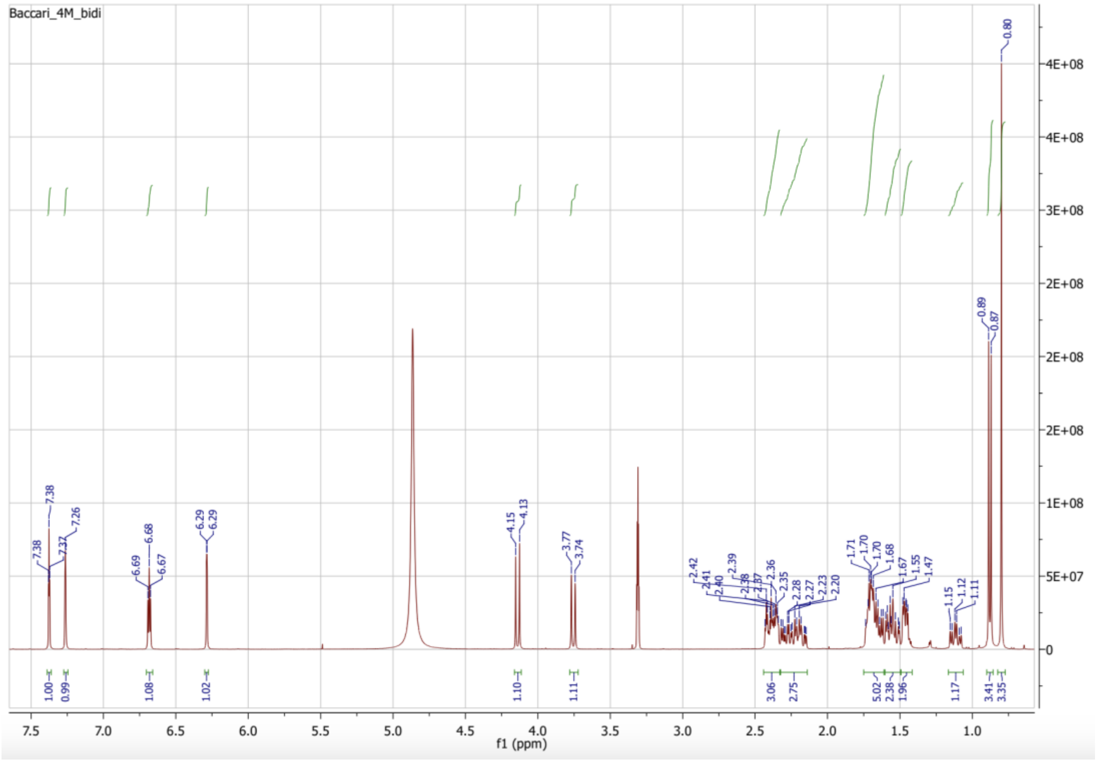

Figure S3: ^{1}H NMR spectrum for Fraction 4 corresponding to Hautriwaic acid.

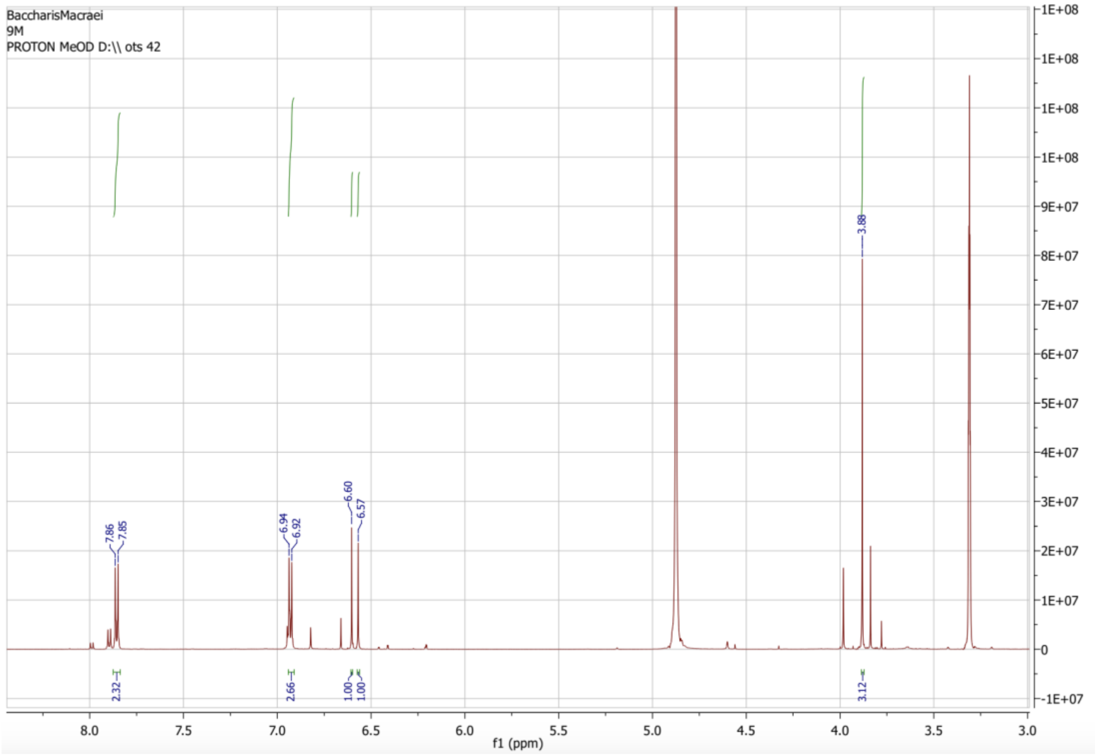

Figure S4: ^{1}H NMR spectrum for Fraction 9 corresponding to Hispidulin.

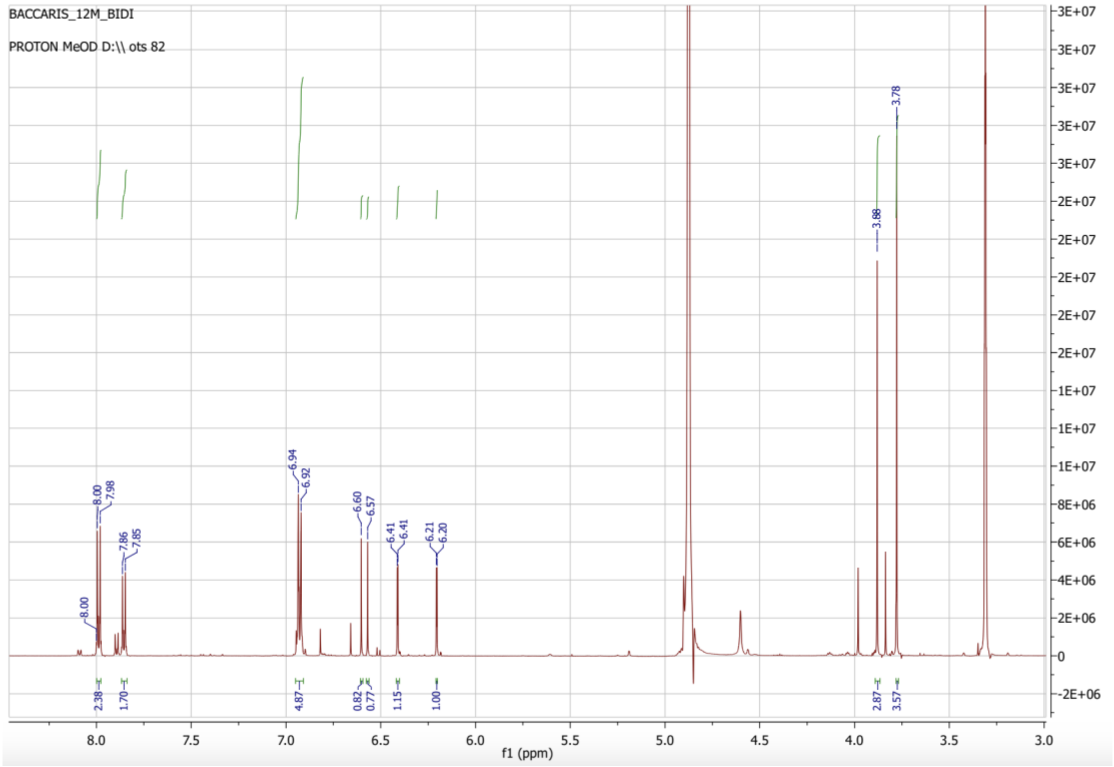

Figure S5: ^{1}H NMR spectrum for Fraction 12 corresponding to mixture of Hispidulin-Isokaempferida.

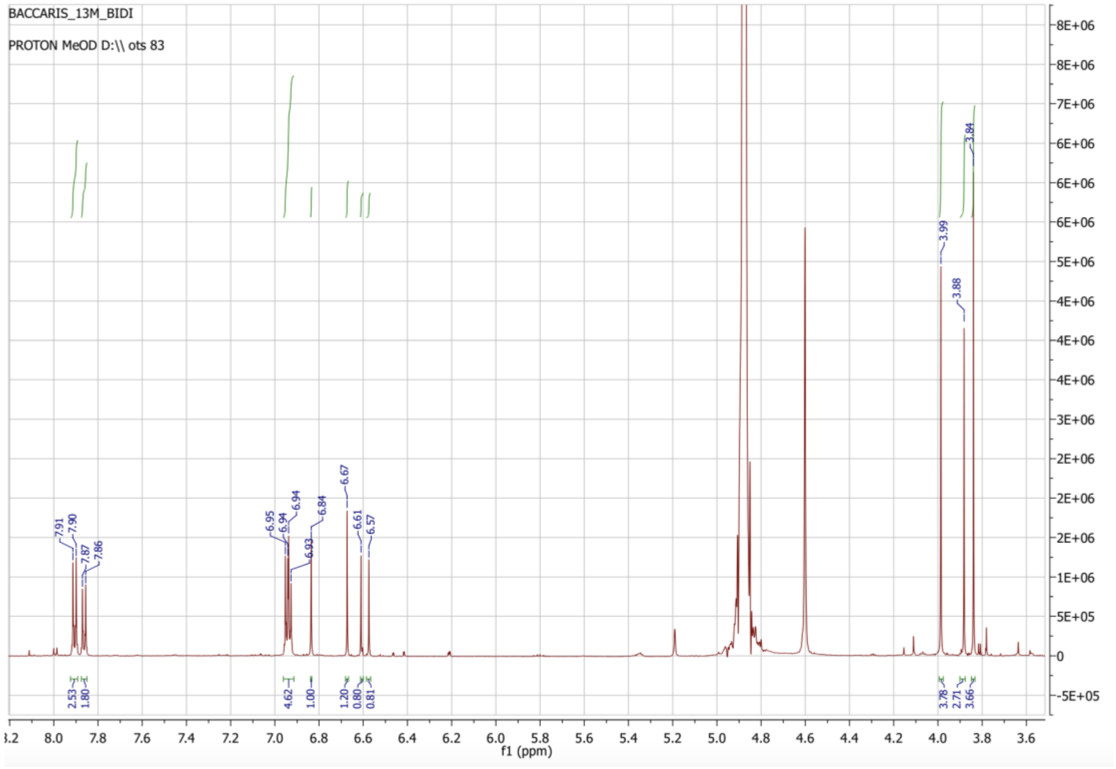

Figure S6: ^{1}H NMR spectrum for Fraction 13 corresponding to mixture of Hispidulin-Cirsimaritina.

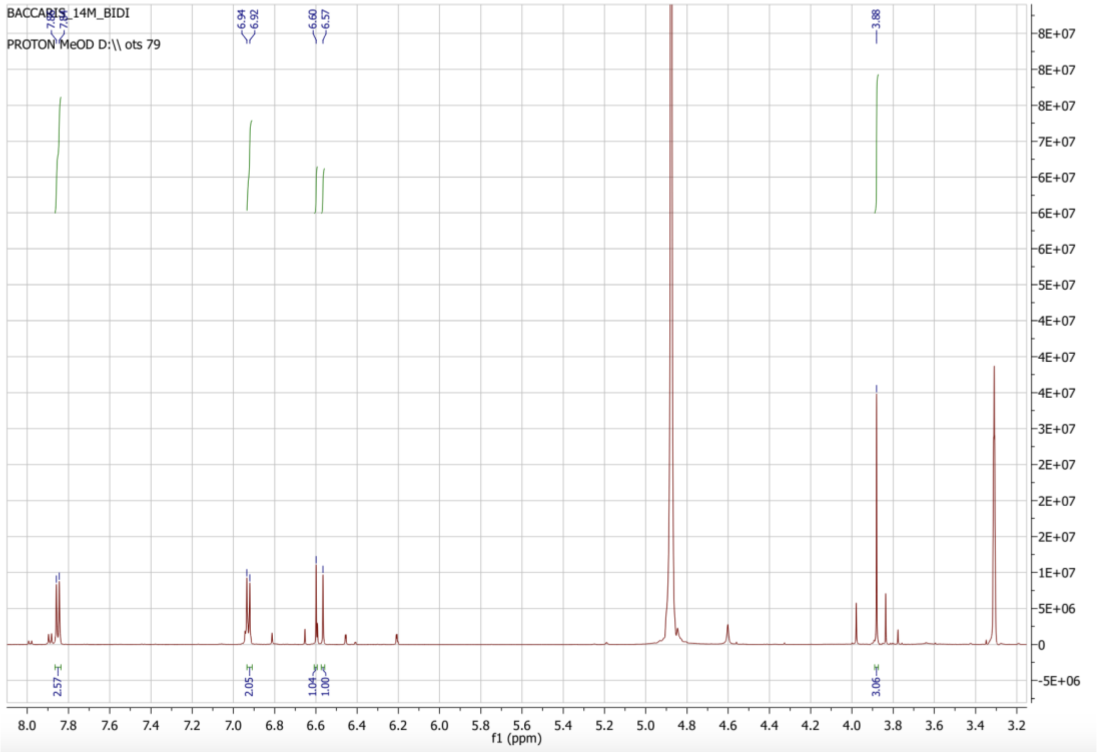

Figure S7: ^{1}H NMR spectrum for Fraction 13 corresponding to Hispidulin.
